# Invasive coronary imaging of inflammation to further characterize high-risk lesions: what options do we have?

**DOI:** 10.3389/fcvm.2024.1352025

**Published:** 2024-02-01

**Authors:** Jonathan Los, Frans B. Mensink, Niekbachsh Mohammadnia, Tjerk S. J. Opstal, Peter Damman, Rick H. J. A. Volleberg, Denise A. M. Peeters, Niels van Royen, Hector M. Garcia-Garcia, Jan H. Cornel, Saloua El Messaoudi, Robert-Jan M. van Geuns

**Affiliations:** ^1^ Department of Cardiology, Radboud University Medical Center, Nijmegen, Netherlands; ^2^ Department of Cardiology, Northwest Clinics, Alkmaar, Netherlands; ^3^ Department of Cardiology, MedStar Washington Hospital Center, Washington, DC, United States; ^4^ Dutch Network for Cardiovascular Research (WCN), Utrecht, Netherlands

**Keywords:** atherosclerosis, inflammation, intravascular ultrasound, near-infrared spectroscopy, optical coherence tomography, near-infrared fluorescence imaging

## Abstract

Coronary atherosclerosis remains a leading cause of morbidity and mortality worldwide. The underlying pathophysiology includes a complex interplay of endothelial dysfunction, lipid accumulation and inflammatory pathways. Multiple structural and inflammatory features of the atherosclerotic lesions have become targets to identify high-risk lesions. Various intracoronary imaging devices have been developed to assess the morphological, biocompositional and molecular profile of the intracoronary atheromata. These techniques guide interventional and therapeutical management and allow the identification and stratification of atherosclerotic lesions. We sought to provide an overview of the inflammatory pathobiology of atherosclerosis, distinct high-risk plaque features and the ability to visualize this process with contemporary intracoronary imaging techniques.

## 1 Introduction

Atherosclerotic cardiovascular disease continues to be a major health burden worldwide (1). Ischemic heart disease is responsible for more than 15% of all global deaths (2, 3). The coronary vessel wall experiences accumulation of inflammatory cells, lipids, fibrous tissue and calcium leading to progressive narrowing of its lumen (4–6). Rupture of the fibrous cap or plaque erosion can trigger local thrombosis, which extends into the coronary lumen and subsequently impedes blood flow (7, 8). Plaque rupture is the most common mechanism of fatal acute myocardial infarction and sudden cardiac death (9). To reduce cardiovascular events, lipid-lowering- and plaque stabilization therapies, including statins and ezetimibe, have become cornerstones in treatment strategy. More recently, Proprotein Convertase Subtilisin Kexin type 9 (PCSK9) monoclonal antibodies have been added to the treatment possibilities (10, 11). Despite these intensive lipid-lowering treatments, residual risks persists. This most likely reflects mechanisms in the biology of atherosclerosis that are incompletely managed by controlling dyslipidemia, which includes the inflammatory response (12, 13). As exemplification, patients with target concentrations of low-density-lipoprotein (LDL) below 1.8 mmol/L and a high-sensitivity C-reactive protein (hsCRP) < 2 mg/L have the best clinical outcomes (14, 15). Furthermore, the anti-inflammatory drugs canakinumab and colchicine have demonstrated to reduce recurrent cardiovascular events (16, 17). Detection of coronary artery wall inflammation might identify which patients benefit from anti-inflammatory therapy (18).

Intracoronary imaging has greatly improved our understanding of the pathophysiology in atherosclerotic cardiovascular disease (19). Most articles discussing intracoronary imaging, focus on the structural characteristics of high-risk plaque. In addition to structural features, the present review aims to address the underlying inflammatory process and whether it is feasible to visualize markers of this process with current and future invasive *in vivo* imaging.

## 2 Inflammation in atherosclerosis

**Figure 1:**
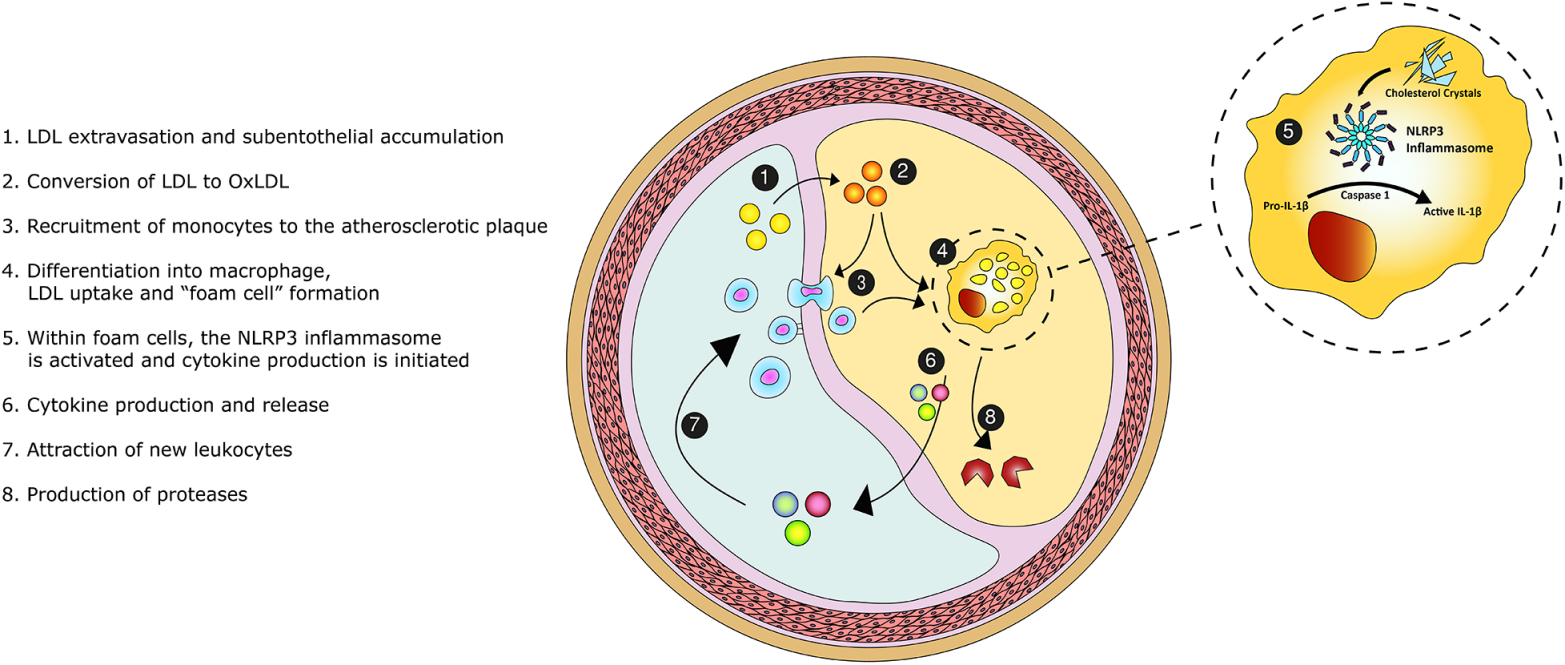
Graphical overview of the inflammatory pathobiology of atherosclerosis. Legend: Low-density-lipoprotein (LDL) enters the intimal space. The accumulated LDL particles become oxidized and promote the recruitment of monocytes. Monocytes transform into macrophages. Upon the scavenging of lipids, macrophage differentiate into foam cells. Within foam cells, the NOD-, LRR- and pyrin domain-containing protein 3 (NLRP3) inflammasome becomes activated and initiates cytokine en chemokine production, perpetuating the inflammatory process. Foam cells secrete proteases causing proteolysis of the extracellular matrix and destabilizing the atherosclerotic plaque.

Atherosclerosis is initiated at the inner layer of the intima (Figure 1). LDL particles accumulate in the subendothelial space at sites with endothelial dysfunction and turbulent flow (20). Subsequently, LDL particles cluster and become oxidized by reactive oxygen species. Oxidized LDL increases local endothelial permeability (21). As a result, patrolling monocytes enter the subintimal space and differentiate into macrophages. These macrophages amass lipids through cholesterol uptake by scavenger receptors, leading to “foam cell” formation. Foam cells induce chemokine and cytokine production, which attracts new leukocytes (22). This positive feedback loop perpetuates and increases plaque formation. The accumulated inflammatory cells secrete a wide range of proteases. Cathepsins and matrix metalloproteinases are common, macrophage-derived, proteases within the atherosclerotic plaque. These proteases are involved in proteolysis of the extracellular matrix, lesion progression and plaque instability (23, 24).

In addition to inflammatory cells, vascular smooth muscle cells have shown to migrate into the intima (20). Upon exposure to lipids and cytokines, vascular smooth muscle cells transform into a proliferating cell type, expressing markers of macrophages (25). These macrophage-like cells can also take up lipids and may promote inflammation (20, 26).

Within the monocytes and macrophages, the NOD-, LRR- and pyrin domain-containing protein 3 (NLRP3) inflammasome pathway initiates cytokine production (27, 28). This protein complex is activated by cholesterol crystals (29, 30). Upon activation, Caspase 1 cleaves the inactive interleukin-1β (IL-1β) precursor. Thereafter, activated IL-1β is released into the circulation. IL-1β induces the inflammatory function of human endothelial cells and stimulates adhesion molecules that recruit leukocytes. IL-1β triggers the release of multiple cytokines, chemokines and other inflammatory mediators (31). For example, interleukin-6 is a downstream cytokine which induces the production of C-reactive protein (CRP) and fibrinogen, promoting thrombosis (28). Therefore, IL-1β is perceived to be the pivotal cytokine in the inflammatory cascade and a driver of atherosclerosis (32). Our understanding of atherosclerosis has thereby evolved to a complex, cholesterol crystal-induced, inflammation of the arterial wall.

## 3 Morphological features of high-risk plaque

**Figure 2:**
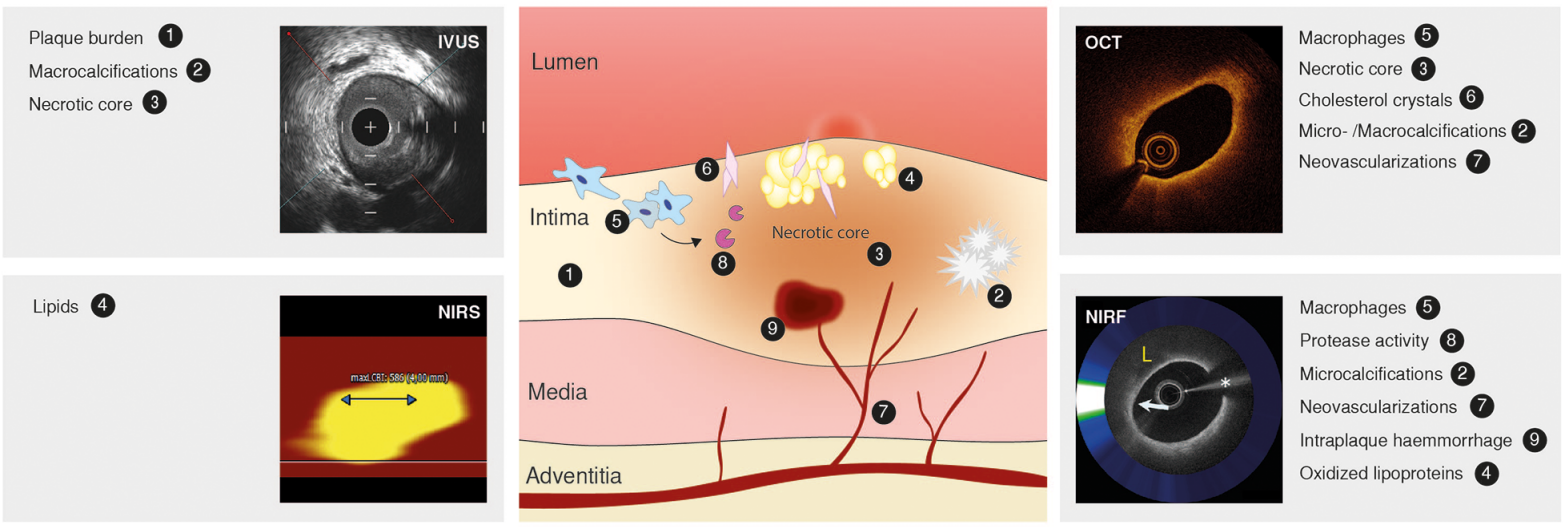
Atherosclerotic lesion, markers of inflammation and high-risk plaque and intravascular imaging techniques. Atherosclerotic lesion (middle) depicting the inflammatory pathobiology of atherosclerosis. Contemporary intracoronary imaging tools (intravascular ultrasound, IVUS; near-infrared spectroscopy, NIRS; optical coherence tomography, OCT; near-infrared fluorescence, NIRF) and their ability to display the process. Image of NIRF signal reused with permission from Ughi et al. (122).

Identification of high-risk lesions is of great importance, given that most atherosclerotic plaques responsible for acute coronary syndromes (ACS) are angiographically mild (33, 34). High-risk plaque refers to a lesion at high short-term risk of causing an acute clinical event (5). Lipid pools, cholesterol crystals, presence of macrophage, a large necrotic core, intraplaque hemorrhage and microcalcifications have been identified as hallmarks for high-risk lesions and represent markers of the underlying inflammatory driven process of atherosclerosis (35, 36). These hallmarks have become targets of intracoronary imaging techniques (Figure 2).

### 3.1 Lipid pools and cholesterol crystals

Plasma-derived lipids accumulate in the subintimal space in the initial phase of plaque formation (20). Lipid pools do not only initiate an inflammatory reaction, but also increase biomechanical stress (37). Liquid cholesterol in these pools crystalizes, leading to volume expansion (38). This volume increase destabilizes the atherosclerotic plaque. Moreover, cholesterol crystals can injure the arterial wall and disrupt the plaque (39).

Cholesterol crystals are found in ∼39% of *de novo* culprit lesions of patients with either ACS or chronic coronary syndrome (CCS), and correlate with high-risk morphological features of culprit lesions (40). Cholesterol crystals are more often observed in culprit lesions in ST-elevation ACS patients compared to non-ST-elevation ACS patients, as is an increase in macrophage accumulation, spotty calcifications, mean lipid arc, thin-cap fibroatheromas (TCFAs) and thrombus (41). This supports the hypothesis that cholesterol crystals increase plaque vulnerability and trigger plaque rupture.

### 3.2 Macrophage and necrotic core

The necrotic core results from cell death and the inability to clear this debris. Macrophages play a major role in this process (42). Hypoxia, lipids and oxidative stress have shown to induce apoptosis in different cell types, including leukocytes and vascular smooth muscle cells (43). These signals trigger DNA fragmentation and expression of cell-surface markers that attract phagocytes (44). As macrophages are also the main phagocytes within the atherosclerotic plaque, effective clearance depends on neighboring cells. This process is called efferocytosis (45). There is no inflammatory reaction associated with apoptosis or efferocytosis, as cellular constituents are phagocytosed instead of released in the surroundings (44, 46). Within lipid-laden foam cells, intracellular cholesterol crystallizes and induces apoptosis (39). Efferocytosis becomes insufficient, which leads to a pool of dead macrophage forming a necrotic core.

A TCFA containing a large necrotic core, infiltrated by a high amount of macrophages is often displayed as “a classical example” of a lesion prone for rupture (47–49). The fibrous cap is defined as a distinctive layer of connective tissue overlying the necrotic core. It consists of smooth muscle cells in an extracellular matrix of collagen, proteoglycans and elastin. The media and adjacent adventitia may be infiltrated by varying degrees of lymphocytes, macrophages and foam cells (50, 51). Historically, fibrous caps with a minimum thickness of <65 µm are considered to be thin, as histopathological analysis showed a cap thickness of <64 µm in 95% of arteries with ruptured plaque (49).

### 3.3 Plaque neovascularization and intraplaque hemorrhage

Neovascularization and intraplaque hemorrhage are common phenomenons within atherosclerotic plaques (52, 53). Neovessels are already established in the early phase of the atherosclerotic process and mainly originate from angiogenesis out of the vasa vasorum (53). Such neovessels are thin-walled, more fragile and may function as entrance for erythrocytes, lipids and inflammatory cells (35). As example, neovessel density is more prominent at sites infiltrated by macrophages and lymphocytes (53). Neovessels exhibit inadequate endothelial integrity, making them susceptible for microvascular leakage, which is thought to induce intraplaque hemorrhage (54). Accumulation of erythrocytes is associated with lesion instability and necrotic core expansion (52). Therefore, intraplaque hemorrhage may promote inflammation.

### 3.4 Microcalcifications

Atherosclerotic calcification is initiated within areas of inflammation (55). Serial *in vivo* imaging in apoE^−/−^ mice showed that inflammation precedes osteogenic activity and that the initially formed crystals colocalize with macrophages (56). Proposed mechanisms of calcification include the nucleation of necrotic debris into calcium phosphate crystals, reduced activity of inhibitors of vascular calcification and transdifferentiation of intraplaque vascular smooth muscle cells and circulating hematopoietic stem cells into an osteo-, and chondrogenesis phenotype (55, 57). In turn, calcium phosphate crystals have shown to induce a pro-inflammatory response by macrophages (58). This suggests a positive feedback loop between inflammation and calcification. The calcium phosphate crystals congregate into microcalcifications. These microcalcifications, if present in the fibrous cap, may cause microfractures that could destabilize the atherosclerotic plaque (59). Therefore, presence of microcalcifications may refer to a more vulnerable phase in the progression of atherosclerotic plaque within regions of inflammation. Whereas increasing density is thought to reflect a stabilizing process (55).

## 4 Invasive imaging

### 4.1 Coronary angiography

Invasive coronary angiography has established itself as a reference standard for the assessment of coronary artery disease (60). It provides a two-dimensional representation of the coronary lumina, by injecting contrast media and performing different radiographic projections, with minimal information on the vessel wall. Coronary angiography is able to identify, albeit suboptimally, the presence of calcification and thrombus. Calcified lesions can be recognized as apparent radiopacities before contrast injection (61). Thrombus can be determined by contrast filling defects and intraluminal lucencies on the “luminogram” (62). The application of other intracoronary diagnostic tools offers the opportunity to look beyond luminal dimensions to identify previously indiscernible lesions.

Thermography was introduced in the early 2000s as an alternative tool to detect coronary artery wall inflammation (63). It was suggested that temperature heterogeneity could identify high-risk lesions. “Hot plaque”, was supposed to reflect the higher metabolic rate of inflammatory cells. However, intracoronary thermography could not meet its expectations (63). In vivo experiments showed that intracoronary thermistors could not detect subtle changes in temperature during substantial influence of pressure, cardiac motion and coronary blood flow (64). Thereafter, it fell in oblivion. Notwithstanding, contemporary imaging techniques do have the ability to target inflammation.

### 4.2 Intravascular ultrasound (IVUS)

**Figure 3:**
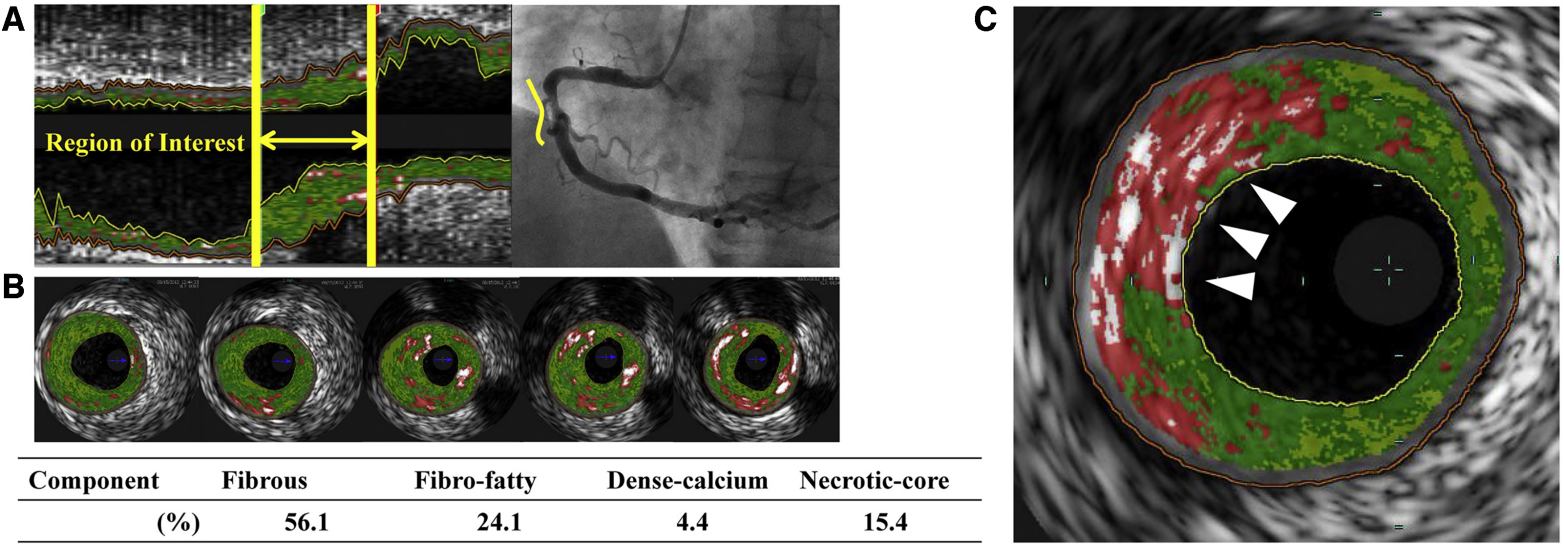
Virtual histology intravascular ultrasound (VH-IVUS) image of atherosclerotic plaque. Based on the reflected ultrasound signals, VH-IVUS automatically provides a colorized tissue map of plaque composition: fibrous (dark green), fibrofatty (light green), calcified (white) and necrotic core (red). (**A**) Longitudinal VH-IVUS image of an atherosclerotic lesion in the right coronary artery. (**B**) Cross-sectional images within the region of interest showing different tissue compositions. (**C**) A thin-cap fibroatheroma (TCFA), characterized as a necrotic-core rich lesion without a clear overlying fibrous cap (arrows). Reused with permission from Kuroda et al. (126).

Novel high-definition IVUS may reach an axial resolution of approximately 40–60 µm using high-frequency ultrasound signals (60 MHz) (65). IVUS can differentiate between various plaque components, since calcified plaques are brighter with acoustic shadowing, while lipid-rich plaques appear less echo dense. Furthermore, IVUS can evaluate serial changes in coronary atheroma, to measure for example the effect of statins or PCSK9 inhibitors on atheroma volume (66–69). Using spectral analysis of the “backscattered” ultrasound signals, IVUS offers the opportunity to estimate plaque composition, so-called virtual histology IVUS (VH-IVUS). VH-IVUS has been used to differentiate fibrous plaque, fibrofatty plaque, necrotic core and calcium (Figure 3), which correlates with histologic samples (70). Moreover, serial changes in plaque morphology and pharmacological effects can be identified with VH-IVUS. For example, the “Integrated Biomarker and Imaging Study 2” (IBIS-2) trial showed that darapladib, a “lipoprotein-associated phospholipase A2 inhibitor”, prevented necrotic core expansion after 12 months (71). However, not all studies could confirm the accuracy of VH-IVUS. Necrotic core determined by VH-IVUS did not correlate with histology within a porcine model (72). Therefore, concerns about the validity remain.

IVUS is less suited to detect microcytic or molecular factors of inflammation, such as macrophage accumulation or cholesterol crystals, owing to its relatively low resolution (73). However, high-risk plaque features detected by IVUS seem to be positively correlated with circulating inflammatory biomarkers, reflecting higher inflammatory activity (74–77). Within the “European Collaborative Project on Inflammation and Vascular Wall Remodeling in Atherosclerosis—Intravascular Ultrasound” (ATHEROREMO-IVUS) study, higher plaque burden and TCFA lesions were associated with higher levels of circulating tumor necrosis factor alpha and lower levels of circulating interleukin-10 (74). Furthermore, lesions with a plaque burden of ≥70% or TCFA were independently associated with higher rate of major adverse cardiac events within the same study (78).

In addition to tissue characterization, mechanical stress can affect coronary arteries (79). It is feasible that plaque rupture occurs at a location subject to higher mechanical stress. Mechanical strain refers to the tensile stress caused by the pulsatile intravascular blood pressure, whereas wall shear stress results from the tangential component of shearing deformation from blood flow (79). Mechanical strain can be assessed by using the displacement of radiofrequent IVUS signals at two different intracoronary pressures. This technique is called palpography (80). Patients presenting with ACS have more high strain spots than patients with CCS (80). Furthermore, the number of high strain spots seemed positively correlated with levels of hsCRP (80). Another *in vivo* study using Yucatan minipigs showed that regions with high strain levels were associated with presence of macrophage (81). However, within “The Providing Regional Observations to Study Predictors of Events in the Coronary Tree” (PROSPECT) trial, no difference was found in strain values between thin- and thick-cap fibroatheroma. They could not confirm the correlation between high strain spots and hsCRP in humans (82). Therefore, the diagnostic value of palpography remains uncertain.

Although smaller in magnitude than mechanical strain, wall shear stress is receiving increasing attention because of its biomechanical relevance. Low wall shear stress acts as a pro-inflammatory and pro-atherogenic stimulus on endothelial cells (79). A three-dimensional reconstruction of the coronary artery lumen is required, which also can be obtained using coronary angiography in combination with IVUS or optical coherence tomography (OCT). Thereafter, wall shear stress maps can be constructed using coronary geometries and computational fluid dynamics calculations (79). Unfortunately, results from *in vivo* studies on the correlation of wall shear stress and plaque progression remain scarce and conflicting. Both low and high wall shear stress have been associated with atherosclerosis and inflammation (79). Therefore, more clinical studies are needed to explore its use.

### 4.3 Near-infrared spectroscopy (NIRS)

IVUS can be combined with near-infrared spectroscopy (NIRS), which projects near-infrared light on the coronary wall, after which the reflected light is analyzed. Since cholesterol has unique features in the wavelength region, NIRS can be applied to characterize lipid-rich plaque, expressed by the lipid core burden index (LCBI) (83, 84). This index is calculated as the number of pixels with a probability of lipid core plaque > 0.6 divided by the total number of pixels and multiplied by 1,000. The MaxLCBI_4mm_ is often used to detect the presence of a large lipid pool, which is the maximum LCBI value for any 4-mm segment (85). The PROSPECT II study showed that highly lipidic lesions with a MaxLCBI_4mm_ ≥ 324.7 were independently associated with future cardiac events (86). Moreover, risk of non-culprit major adverse cardiovascular events (MACE) increased significantly for each 100-unit increase in MaxLCBI_4mm_ in the “Lipid Rich Plaque” (LRP) study (87). NIRS confirms the crucial role lipids fulfil in the development of cardiovascular events and NIRS can be used to assess response to medical therapy, primarily lipid-lowering therapies (88). However, NIRS is unable to identify crystallization of cholesterol and lacks the ability to differentiate between inflamed or non-inflamed lesions. Furthermore, no association between LCBI and inflammatory biomarkers have been found so far (89).

**Figure 4:**
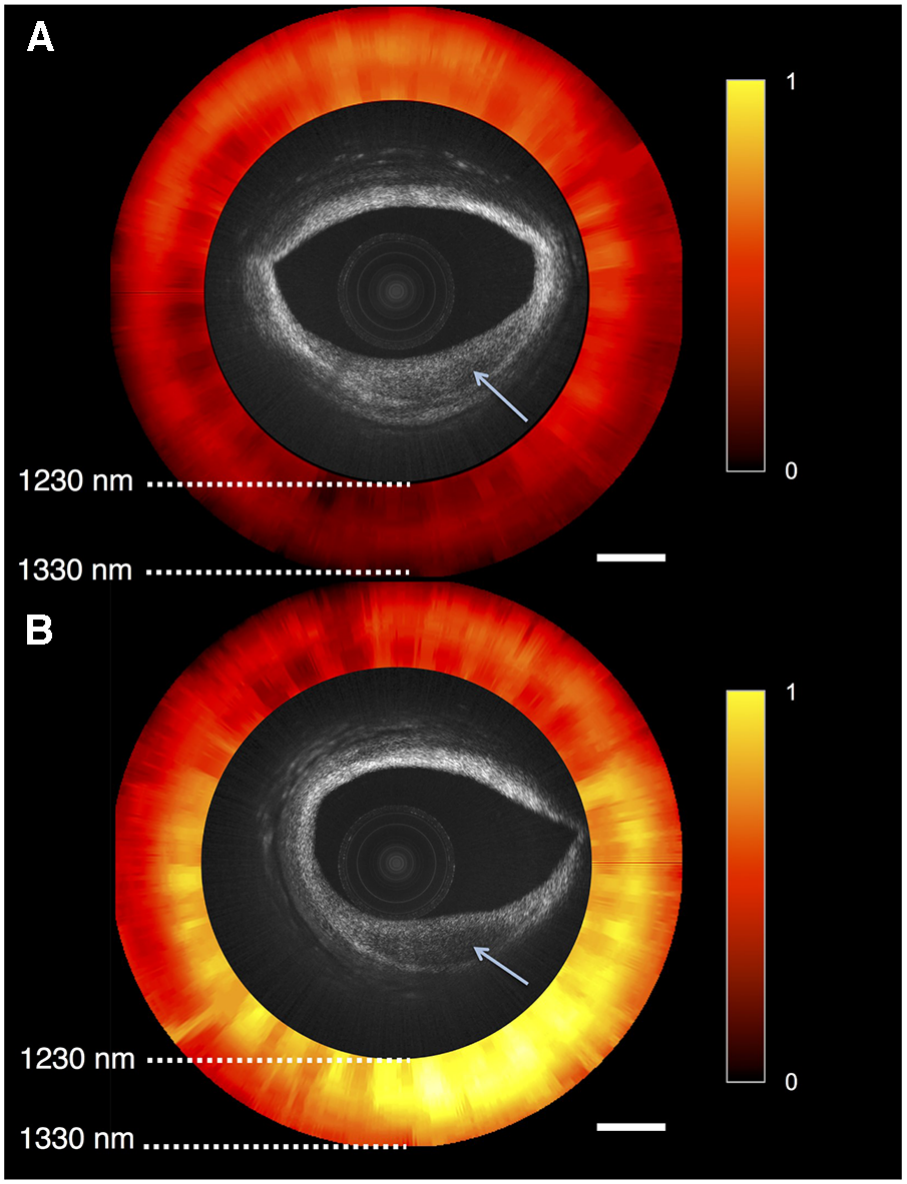
Optical coherence tomography near-infrared spectroscopy (OCT-NIRS) of cadaver coronary artery. Combinded OCT and NIRS (red and yellow circle) imaging of 2 lesions of cadaver coronary artery, showing reduced backscattering (arrows). (**A**) Lesion with low amounts of lipids, compatible with fibrotic tissue, as it is is shown in red. (**B**) Lipid-rich plaque displayed as yellow area. Reprinted with permission from Hoang et al. (90) © The Optical Society.

A novel OCT-NIRS catheter is being developed to provide simultaneous microstructural and compositional imaging (Figure 4) (90). The superior resolution and characteristics of OCT could overcome some limitations inherent to IVUS imaging, as discussed in the next paragraph. A first-in-human study using OCT-NIRS is ongoing (NCT05241665).

### 4.4 Optical coherence tomography (OCT)

**Figure 5:**
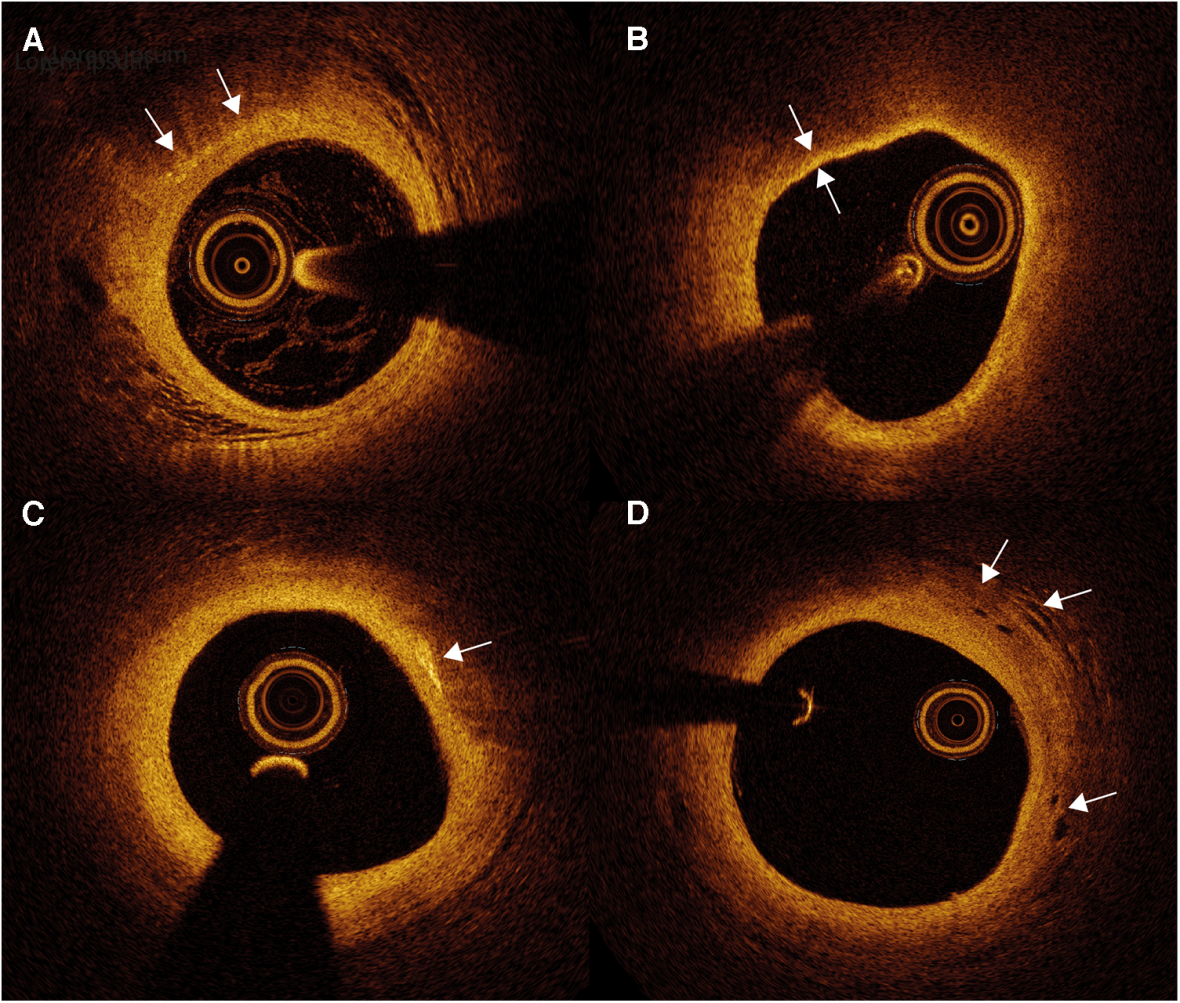
Optical coherence tomography (OCT) images of atherosclerotic plaque and inflammation markers. (**A**) Macrophages appear as signal-rich bright spots with a signal-poor region below (arrows). (**B**) Thin-cap fibroatheroma are identified as an atherosclerotic plaque covered by a fibrous cap of <65 µm (between arrows). (**C**) Cholesterol crystals appear as thin and linear structures with high signal intensity (arrow), often localized nearby lipid-rich plaque. (**D**) Intraplaque neovessels can be identified as well delineated signal-poor voids (arrows).

OCT can provide microstructural images up to a maximal axial resolution of 10 µm at the cost of penetration depth, compared with IVUS (1–2 mm vs. 10 mm). The high resolution not only enables more detailed visualization of calcified nodules, thrombi, TCFAs, plaque erosions and ruptures, but also cholesterol crystal accumulation, macrophages and microvessels (Figure 5) (91, 92).

Macrophages scatter light efficiently, which creates signal-rich regions called bright spots with a cast shadow behind (93). (Figure 5A) While OCT has not been proven to distinguish between active and inactive macrophages (94), bright spots do have a strong correlation with inflammation measured by hsCRP (95). Bright spot density is significantly higher in lipid plaques compared to fibrous plaques and plaques with TCFA show a trend toward higher bright spot density (95). Moreover, bright spot density is also significantly higher in plaques with rupture than those without (95). These findings imply the ability of OCT to provide an overall estimate of macrophage accumulation. Furthermore, presence of OCT-defined macrophage accumulation is associated with adverse clinical outcome. In the “Relationship Between OCT Coronary Plaque Morphology and Clinical Outcome” (CLIMA) study, presence of macrophage accumulation in native left anterior descending coronary artery was associated with more clinical events, especially in the copresence of other high-risk plaque features including a thin fibrous cap and a large lipid arc (96).

Cholesterol crystals appear as thin, linear structures with high signal intensity, often localized nearby lipid-rich plaque (Figure 5C). It is suggested that needle-shaped cholesterol crystals could perforate the fibrous cap, causing plaque instability (93).

Intraplaque neovessels can be identified as well delineated signal-poor voids, which can be followed in consecutive frames (Figure 5D) (93). An ex vivo OCT study showed that coronary atherosclerotic plaques with neovessels were accompanied with greater luminal narrowing (97). Subsequent neovessel rupture could induce intraplaque hemorrhage. However, studies about neovessels and neovessel rupture on OCT imaging remain scarce.

OCT is the only imaging modality with sufficient spatial resolution to adequately measure fibrous cap thickness (Figure 5B). Numerous prospective and retrospective studies have demonstrated an association between OCT-identified TCFA and clinical outcome, whether or not in combination with other features of plaque instability (96, 98). The fibrous cap thickness cutoff to define TCFA differs between studies, as it has been suggested that the 65 um cutoff obtained in histopathological studies should be enlarged to adjust for potential tissue shrinkage during histopathological tissue processing (93). Nevertheless, Jiang et al. found a similar optimal cutoff of 66.7 um to distinguish lesions at higher risk of causing events (98). In this study, 883 patients were included, all 3 main epicardial vessels were scanned and follow-up lasted up to 4 years. OCT can differentiate whether ACS arises from rupture of the fibrous cap or endothelial injury with an intact fibrous cap, i.e. plaque erosion. These distinct patterns might have different underlying pathobiologies. The presence of OCT-identified culprit plaque rupture is associated with lower levels of T-cells but higher levels of effector molecules involved in the innate immune response compared to ACS with intact fibrous caps (99). This may indicate that the adaptive immune system plays an important role in inducing endothelial erosion. In proteomics analysis, patients with ruptured plaques also had a higher inflammatory response and more MACE during 2 years of follow-up (100).

OCT allows to evaluate change in high-risk plaque characteristics (101). The “High-Resolution Assessment of Coronary Plaques in a Global Evolocumab Randomized Study” (HUYGENS) showed that intensive lipid-lowering therapy with high-dose statins and evolocumab increased minimum fibrous cap thickness and decreased the macrophage index on serial OCT. The combination of statin and evolocumab resulted in more favorable changes than statin therapy alone (102).

#### 4.4.1 Micro optical coherence tomography (µOCT)

In 2011, micro-OCT (*μ*OCT) was introduced to improve the resolution of OCT imaging systems to achieve an axial resolution of 1–2 μm, which is another ten-fold improvement (103). Therefore, μOCT is capable of visualizing independent cells and subcellular features. Moreover, μOCT is able to differentiate between multiple inflammatory cells, including leukocytes, monocytes and macrophages.

An intravascular μOCT catheter suitable for *in vivo* imaging was recently introduced. This device is able to display a wide range of cells and subcellular structures, including leukocytes, macrophages, smooth muscle cells, cholesterol crystals and platelets within rabbit aortae *in vivo* and human cadaver coronary arteries (104). To acquire high-resolution images, current μOCT imaging systems emit light with 800 nm wavelength. The use of a shorter wavelength compared to standard OCT might be at the expense of penetration depth, which is already a disadvantage compared to IVUS (105). Nevertheless, μOCT has the potential to visualize local inflammatory processes *in vivo* such as leukocyte adhesion, foam cell formation or inflammatory cells surrounding cholesterol crystals on microscopic level. More clinical studies are needed to explore further utilities.

### 4.5 Near-infrared fluorescence (NIRF)

Near-infrared fluorescence (NIRF) imaging is an emerging technique, allowing the visualization of molecular processes within the atherosclerotic plaque. It uses imaging agents which bind to specific targets, including protease activity, LDL, fibrin deposition and microcalcifications (106–108). These imaging agents consist of an fluorochrome, conjugated to an antibody, molecule or peptide. After injection, a NIRF-catheter is advanced within the coronary artery. A continuous wave laser diode emits excitational light within a 650 to 950 nm window (NIR spectral region) to stimulate the fluorophores. The subsequent fluorescence emission is collected and filtered within the NIRF-catheter (109).

Protease-activatable fluorophores have been developed to visualize enzymatic activity. At baseline, fluorophores emittance is quenched, but increases significantly after cleavage (110). Enzymatically active cathepsins, detected by NIRF, seem to colocalize with cathepsins and macrophages on immunohistochemistry in animal and human atheromata (107, 111, 112). Matrix metalloproteinase-specific fluorophores have shown similar results on NIRF imaging (113, 114). Indocyanine green (ICG) is an imaging agent, which can directly visualize macrophages. After injection, ICG is internalized by macrophages and foam cells by binding to albumin or LDL (108). In a recent study, the ICG NIRF signal, measured in freshly isolated carotid plaques, was highest in the most stenotic area. Subsequent histopathological analyses established that ICG targeted endothelial abnormalities, such as disrupted fibrous caps and areas of neovascularization. ICG concentrated on zones of plaque lipids, macrophages and intraplaque hemorrhage (115). Furthermore, fluorophores have been developed to target fibrin deposition, activated factor XIIIa or thrombin activity to assess thrombosis. Validation of these fluorophores mostly rely on non-invasive NIRF imaging techniques (116–118). However, intravascular NIRF has been able to detect fibrin deposition overlying stent struts in rabbits (119).

**Figure 6:**
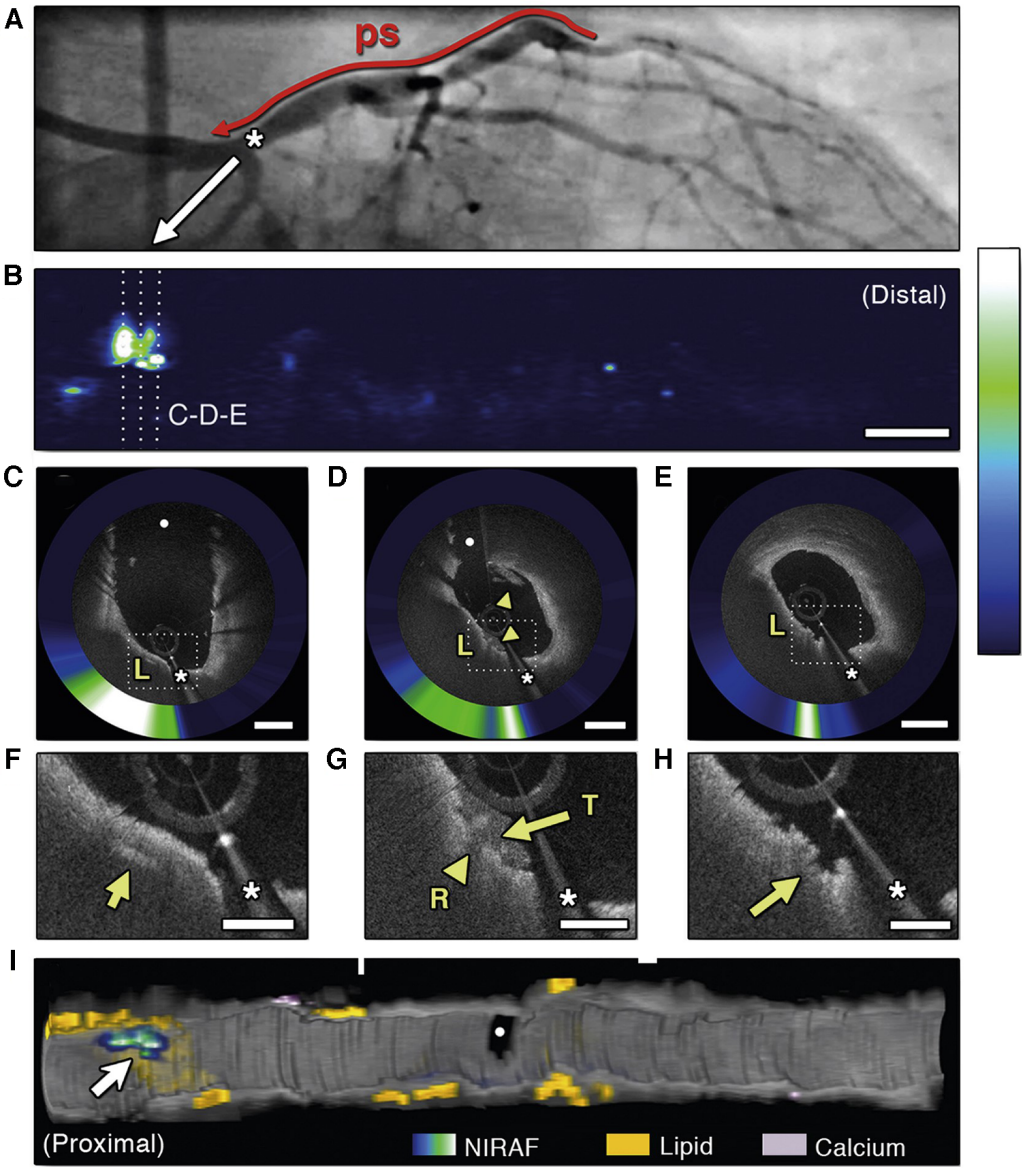
Optical coherence tomography near-infrared autofluorescence (OCT-NIRAF) imaging of a ruptured thin-cap fibroatheroma. (**A**) Coronary angiography of the left circumflex coronary artery. (**B**) 2-dimensional map of NIRAF signal. (**C–E**) Cross sectional OCT—NIRAF images showing a rupture of a thin fibrous cap covered with a small thrombus. The rupture site shows high NIRAF. (**F,G**) Magnified views revealing a cholesterol crystal (**F**, arrow), thrombus (**G**, arrows) and the rupture site (**H**, arrow). All colocalized with elevated NIRAF. (**I**) 3-dimensional rendered map demonstrating that the high NIRAF signal appears within regions containing high amount of lipids (arrow). The asterisk (*) corresponds with catheter artefact. L, lipid; R, rupture site; T, thrombus. Reused with permission from Ughi et al. (122).

Hybridization of intravascular molecular and structural imaging could potentially allow further study of the pathophysiological mechanisms of arterial plaques (120, 121). Both NIRF-OCT and NIRF-IVUS are being developed. The dual-of modality OCT and NIRF, can detect fluorescence from naturally occurring molecules, called near-infrared autofluorescence (NIRAF). NIRAF is elevated in advanced necrotic core-containing lesions and is associated with a high-risk morphological plaque phenotype (Figure 6) (122, 123). Interestingly, NIRAF elevation is specific to plaques with macrophage accumulations, as shown by OCT (122). However, the converse is not true, since many areas with elevated macrophage accumulation on OCT were NIRAF negative. This could be explained by low sensitivity/high specificity of NIRAF to macrophage accumulation, or by the concept of different macrophage phenotypes. The underlying molecular and chemical mechanisms that produce NIRAF are not yet fully understood.

## 5 Discussion

Atherosclerotic cardiovascular disease is a complex chronic inflammatory and fibroproliferative process fueled by atherogenic lipoproteins. This implies the requirement of precise diagnostic tools and targeted treatment strategies (124). Systemic inflammation has emerged as a therapeutic target to reduce cardiovascular events (16, 17). Intracoronary imaging allows the judgement of disease state of atheromata and identification of high-risk lesions. Given the inherent characteristics of different imaging modalities, they all facilitate distinctive insights in the inflammatory pathobiology of atherosclerosis (Table 1). IVUS gives a good “overview” of plaque burden and plaque composition. A higher plaque burden is associated with elevated systemic inflammation, reflected by increased pro-inflammatory biomarkers. However, it is unable to directly visualize the inflammatory process. NIRS provides a chemical analysis of the arterial wall but lacks the ability to detect inflammatory markers or cholesterol crystals. OCT is able to detect and measure TCFA, macrophages, neovessels and cholesterol crystals. Moreover, additional increase of resolution with µOCT allows further detection of individual cells and subcellular substances. NIRF imaging displays molecular- and inflammatory activity by targeting specific molecules, thereby allowing detection of early- and advanced stages of atherosclerosis.

**Table 1:** Characteristics of the imaging modalities and their ability to display high-risk features and inflammation markers.

	(VH) IVUS	NIRS	(µ)OCT	NIRF
Technique	Ultrasound	Near-infrared	Infrared	Near-infrared
Requiring blood removal	−	−	+	−
Axial resolution	up to 40–60 µm (HD IVUS)	NA	10 µm (OCT), 1–2 mm (µOCT)	NA
Penetration depth	10 mm	<3 mm	1–2 mm	3 mm
Quantification of plaque burden	+++	−	+	−
Lipids	++	+++	++	+++
Cholesterol crystals	−	−	+	−
Macrophage	−	−	++ (quantification, plus differentiation for µOCT)	+++ (activity)
Necrotic core	+	−	++	−
Detection of TCFA (<65 µm)	+	−	+++	−
Neovessels	−	−	+	+
Intraplaque hemorrhage	−	−	−	+
Microcalcifications	+	−	++	+++
Suitable (clinical) settings for use	– Assessment of vessel- and lumen dimensions, plaque morphology and aorto-ostial junction – Identification of high-risk lesions based on high plaque burden and small minimum lumen area – Guidance of percutaneous coronary intervention	– Detection of lipid-rich plaque – Combined with IVUS with similar indications	– Detailed assessment of plaque morphology and lumen dimensions – Detection of thrombus, plaque rupture and plaque erosion in unclear ACS mechanism – Identification of high-risk lesions based on TCFA – Guidance of percutaneous coronary intervention	– Detection of specific target molecules – Combined with OCT with similar indications

(VH) IVUS, (virtual histology) intravascular ultrasound; NIRS, near-infrared spectroscopy; (µ)OCT, (micro) optical coherence tomography; NIRF, near-infrared fluorescence. −, not possible; +,adequate; ++, good; +++, excellent; NA, not applicable; TCFA, thin-cap fibroatheroma; HD, high-definition; ACS, acute coronary syndrome.

Lesions with high-risk features on intracoronary imaging have shown to be predictive of MACE (125). However, current positive predictive value is still moderate. Novel hybrid modalities, in particular NIRF-OCT, could provide complementary morphological and functional imaging, further improving the diagnostic performance and prognostic stratification. In the near future, they may identify high-risk lesions of clinical value to revascularize or optimize medical therapy. For the moment, invasive imaging mainly has clinical indications (Table 1), but they can simultaneously identify more specific inflammatory characteristics, which strengthens the case for inflammation-targeted therapies. Moreover, hallmarks of inflammation and high-risk plaque are useful surrogate endpoints to assess the potency of medical therapy.

## 6 Conclusion

Contemporary and future intracoronary imaging techniques allow the identification of inflammatory markers within atherosclerotic plaque. They assess the biochemical composition and the underlying pathophysiology. Furthermore, they serve as a mechanism to evaluate drug efficacy. Conscientious implementation may allow the development of patient tailored treatment strategies and improve patient outcome.
